# Numerical Characterization for Electrical Conductivity of Two-Dimensional Nanocomposite Systems with Conducting Fiber Fillers

**DOI:** 10.3390/ma13102410

**Published:** 2020-05-24

**Authors:** Jungmin Lee, Yesol Yun, Sang Hyun Lee, Jinyoung Hwang

**Affiliations:** 1 School of Electronics and Information Engineering, Korea Aerospace University, Goyang-si 10540, Korea; jeungmin95@kau.ac.kr; 2 School of Electrical Engineering, Korea University, Seoul 02841, Korea; yesolar@korea.ac.kr

**Keywords:** nano-composites, Monte Carlo simulation, percolation networks, electrical conductivity

## Abstract

Hybrid nanotube composite systems with two different types of fillers attract considerable attention in several applications. The incorporation of secondary fillers exhibits conflicting behaviors of the electrical conductivity, which either increases or decreases according to the dimension of secondary fillers. This paper addresses quantitative models to predict the electrical performance in the configuration of two dimensional systems with one-dimensional secondary fillers. To characterize these properties, Monte Carlo simulations are conducted for percolating networks with a realistic model with the consideration of the resistance of conducting NWs, which conventional computational approaches mostly lack from the common assumption of zero-resistance or perfect conducting NWs. The simulation results with nonperfect conductor NWs are compared with the previous results of perfect conductors. The variation of the electrical conductivity reduces with the consideration of the resistance as compared to the cases with perfect conducting fillers, where the overall electrical conductivity solely originates from the contact resistance caused by tunneling effects between NWs. In addition, it is observed that the resistance associated with the case of invariant conductivity with respect to the dimension of the secondary fillers increases, resulting in the need for secondary fillers with the increased scale to achieve the same electrical performance. The results offer useful design guidelines for the use of a two-dimensional percolation network for flexible conducting electrodes.

## 1. Introduction

Conducting polymer composites have become essential components for various modern electronic applications including wearable sensors, flexible displays, batteries, and solar cells, etc. [1,2,3,4]. In particular, CNT-based composites have recently been studied for specific improved properties in those applications [5,6,7]. The percolation networks of one-dimensional nanostructures, two-dimensional materials, such as graphene, and conducting polymers, such as PEDOT:PSS and polyaniline, are considered for their key enablers. In particular, percolation networks of one-dimensional nanowires (NWs) have been extensively studied for applications of flexible conducting polymers. Ultra-high aspect ratio one-dimensional fillers of highly conductive materials, such as carbon nanotube (CNT), gold (Au), silver (Ag), copper (Cu), and their alloys, are desired to achieve the outstanding structural and electrical performance at low coverage. Among those options, CNT-based polymer composites have been intensively investigated for very attractive options, in particular, of their extremely large aspect ratio and expanded surface area. Furthermore, high-quality NWs can be synthesized via low-cost and scalable wet-based process, and a random NW network can be fabricated by coating a dispersed NW solution on a flexible substrate using a slot-die method, bar-coating, and spin coating, and spray coating process [2,4]. Futhermore, a number of studies have addressed the improvement of the electrical performance of the NW network. CNTs/Cu composites show fascinating electrical and thermal conductivities with a larger value of thermal expansion (CTE) coefficient than copper [8]. CNTs based composites also have been utilized in the design of microscopic flexible electronics, such as high-performance Li-S batteries [9], Li-ion batteries [10], and capacitive strain sensors for human motion detection [11,12]. Furthermore, nitrogen-doped CNT-based composites are composite bifunctional catalysts for improving the performance of the rechargeable zinc-air battery [13]. For device applications, this system can be applied to an electromagnetic shield for light-weight and flexibility, and an alternative for metal conductors that are erosive, heavy-weight, costly.

For additional improvement of the performance, the incorporation of particulate fillers can be considered to form the excluded volume that effectively leads to a segregated CNT network [14,15,16,17,18]. In consequence, the probability of CNTs forming a conducting network increases in the segregated composite system, indicating that much lower CNT loading suffices to reach a certain degree of the electrical conductivity [15]. Thus, as compared to CNT composites made only of CNT fillers, the segregated composite system could effectively relieve the cost issue of CNTs. From a mechanical point of view, dramatic strengthening and toughening, which are essential requisites for practical applications, can be achieved by particulate fillers, such as silica, CaCO${}_{3}$, and BaSO${}_{4}$. It has an effect on creating electrical junctions between CNTs. The incorporation of fillers into CNT-filled polymers can reduce the space available for CNTs to form conductive networks and could increase the conductivity because CNTs cannot diffuse into the fillers which are solid particles [19,20,21,22]. Therefore, CNT-based polymer composites, which are comprised of CNTs and micro-scale or nano-scale secondary fillers, change the electrical conductivity due to the side effect of secondary particulate fillers. Micro-scale fillers increase the electrical conductivity for the excluded volume, while nano-scale secondary fillers decrease the electrical conductivity.

This work addresses the computational characterization of the effect of the dimensional properties of secondary particulate fillers on the electrical conductivity of CNT polymer composites. In the previous research, to clarify the contribution of the size effect of secondary particulate fillers on the electrical conductivity of the nanocomposite network, the perfect conductor is assumed for the electrical property of the CNT filler, i.e., the resistivity of the CNT is set to zero to conduct Monte Carlo (MC) simulation for the prediction of percolating behaviors of the CNT network in CNT/silica composites [23]. Thus, the contact resistance of the CNT network is solely a source of the network sheet resistance. Therefore, this work proposes to evaluate the electrical conductivity of the percolating nanocomposite network with the consideration of the resistivity of CNT materials, i.e., nonperfect conducting materials are used for one-dimensional conducting fillers. In addition, we provide the refined interpretation of the change in the electrical conductivity of the CNT composites with respect to the size of particulate fillers via the combination of Swiss cheese models and the underlying percolation theory with nonperfect conducting fillers. The results allow to predict the electrical performance of nanocomposites in a fast and simplified manner as compared to conventional methods for the same purposes.

The conflicting electrical behaviors caused by the addition of secondary particulate fillers can be briefly described with Swiss cheese model. A random configuration of secondary particulate fillers on a polymer matrix can be modeled in a shape of Swiss cheese with many pores. A pore represents a single secondary filler which excludes conducting fibers, and the volume of “cheese” is the space that conducting fibers can occupy. A network of conducting fillers presents the collection of all conducting paths in the polymer matrix with particulate fillers. The CNT network is separated from the conducting fiber composite to evaluate the effective conductance of conducting paths. The local conductance of a point within the volume of Swiss cheese model depends on the density of CNT fibers placed around the corresponding region. To assess the degree and formation of an electrical network and to calculate the total conductance, an available configuration of the effective conducting network is obtained from the joint consideration of the conducting path and geometry of the particulate fillers. Thus, the total conductance can be evaluated by considering the continuum percolation property of the effective network. The transition of the electrical conductivity is observed in accordance with the excluded volume caused by secondary particles, i.e., the excluded volume improves or prevents the conductivity of CNT composites depending on the particle size. The network morphology of CNT composites depends on the size of the particulate fillers, resulting in the variation of the electrical conductivity. From this model, a numerical characterization of conflicting conducting behaviors is carried out based on a unified framework. Since most of available NW composite networks are created with conducting but nonzero resistance fillers, understanding of the impact on the electrical properties of nonzero resistance fillers is necessary. This work develops quantitative models of the electrical conductivity of two-dimensional NW composite networks with one-dimensional nonperfect conducting fillers via MC based computation. Based on computational models, the change of the network sheet resistance involved with the consideration of the NW resistivity is investigated with respect to various design factors for performance improvement. The results are compared with the perfect conducting NW percolating network. According to the results, the network conductance exhibits a relatively small change with the change of the filler size variation, resulting in the change of linear slopes, which suggests the prediction of the network electrical conductivity with an enhanced accuracy. The corresponding performance is also demonstrated in a computational way.

## 2. Method

The MC simulation is conducted to identify percolating behaviors of the CNT network in CNT/silica composites under various size and concentration conditions of composite components. In order to predict the sheet conductance (σ) of a one-dimensional NW percolation network, a random instance of the NW network is generated in a square domain by placing NWs and particulate fillers. A sample square domain of 25 μm × 25 μm is considered to obtain the CNT network. A single flexible CNT wire in the composites is modeled as a long thin cylindrical object with many joints to reflect the bending of the wire, while silica particles are represented in a circular shape with the diameter equal to an average size of silica particles.

Circular particles are scattered in the two-dimensional domain via random choices of central coordinates of nonoverlapping individual particles. For an accuracy of the quantitative analysis with the total volume of the particulate fillers, all silica particles lie nonoverlapping on the domain. To this end, the center coordinates of individual circular objects are chosen uniformly at random inside the domain. Subsequently, the fillers are placed sequentially so that the distances from the chosen center position to the center positions of all pre-existing spheres are apart by larger than the diameter. The number of particles to be placed in the domain is determined from the ratio of the silica volume to the domain volume.

**Figure 1:**
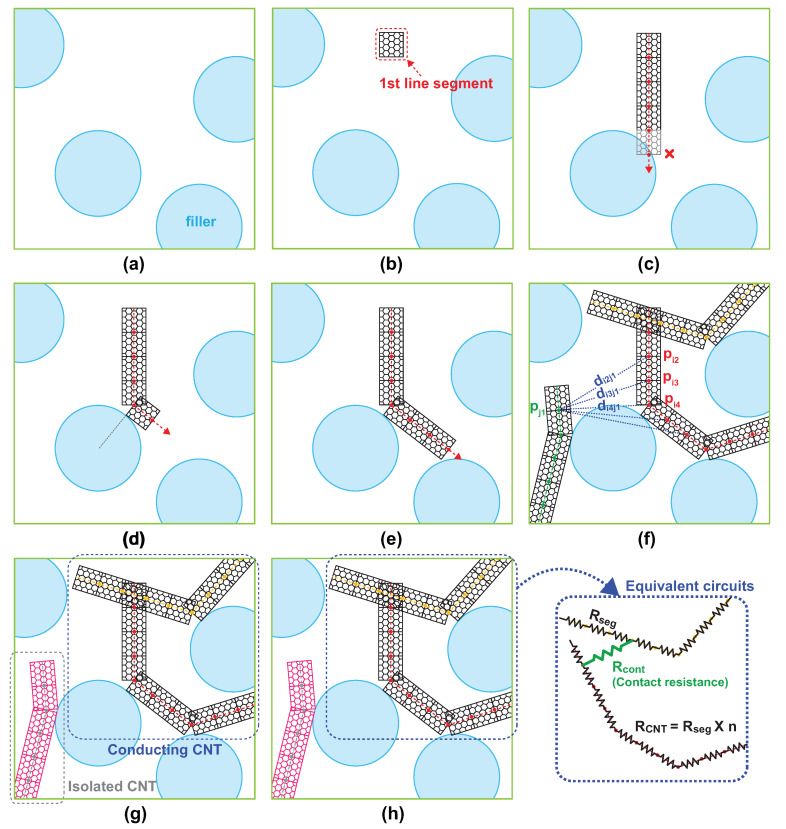
Generation of an instance of CNT/silica composite in Monte Carlo simulation: (**a**) placement of particulate fillers, (**b**–**e**) creation of a single bending conducting filler, (**f**) examination of the connection status of NWs, (**g**) clustering analysis of the NW network, and (**h**) equivalent circuits of the NW network for KCL application.

Upon the completion of the placement of all nonoverlapping particulate fillers, a CNT network is created on the domain with multiple random instances of a flexible CNT wire. A NW is placed uniformly on the simulation domain so that it does not penetrate pre-located particulate fillers but bends to circumvent them. Figure 1 illustrates the generation of a single bendable conducting CNT wire. The flexibility of the NW is reflected by modeling it geometrically as a series of small line segments contiguously connected with joints that are allowed to bend freely within a certain degree, which is chosen as 120${}^{\circ }$ from experimental results [24], such that the width and length of a single segment equal to the diameter and length of a cylindrical NW, respectively. To create a single flexible NW on the simulation domain, constituting line segments are placed in sequence from one end to the other. A random choice of the position of the first line segment is examined if it is placed in the domain that is not occupied by any spherical filler. If the position of the first segment proves valid, a random direction which the NW stretches toward is subsequently chosen. The next line segments are repeatedly placed in a straight line unless touching pre-occupying spheres. If the candidate position of the next segment is predicted to be inside a spherical filler, the line segment bends to change the direction toward a tangential direction of the spherical shape at the contact point to avoid an undesired penetration so that a flexible wire smoothly circumvents the sphere to stretch forward. The length of line segments forming a single flexible wire is set the same as the width of the wire, which facilitates the test for the contact of two flexible wires. Thus, the line segment can be viewed as a square shape with the identical height and width. The test for the contact is sufficient to examine if another line segment from a different NW lies within a spherical ball of radius equal to the length of the line segment. The range of the bending angle of the joint between two consecutive line segments is limited to 120${}^{\circ }$, which is determined by the physical property of a CNT where the carbon tube structure can be preserved with very large bending angle without atomic destruction for its perfect hexagonal structure of a CNT NW. If a CNT/silica composite network is constructed, the connection status of NWs in the CNT network is examined by checking the contact of line segments from all other NWs in the domain. The connectivity between two wires is determined by calculating all possible pairwise distances between line segments belonging to respective wires. If the shortest distance between the two segments is less than the distance of the tunneling effect, the NWs are considered to touch each other. To consider the NW resistance in the network, a novel technique is developed forsimulation. The NW resistance comes into play in the network if a single NW forms a conducting path of the percolating network. This happens when the NW has contacts with at least two different NWs. Since the resistance is proportional to the length of the resistor, the length of the portion in between two contact points of the single NW determines the overall resistance straightforwardly. An efficient way of calculating the distance between two contact points is to count the number of line segments connecting two contact points. While placing individual line segments to create a single NW, the number of line segments is counted to obtain the desired length of the NW. Once a new line segment is found to touch another line segment from a different NW, the index of the corresponding NW is available from the count of line segments. Since the count of line segments between two adjacent contact points, which are obtained by the difference between indices of contact line segments, is proportional to the distance between two contact points. The count of line segments *n* is multiplied by the resistivity of NW ρ and the length of the line segment *w*, which is chosen identical to the width of the NW, to calculate an equivalent resistance given by nρw. Thus, equivalent resistances vary with the distance between contact points. If a single NW has more than two contact points with adjacent NWs, all pairs of two consecutive contact points act as a serial connection of equivalent NW resistors with contact resistance bypasses connected at each junction, as illustrated in Figure 1.

The connectivity information of an individual NW is collected upon the completion of its placement to form an adjacent matrix representing the relationship among NWs. Based on this information, a clustering analysis for the CNT network is conducted via union-find algorithm for exploring percolating clusters that traverse across the square domain. An individual cluster is expanded gradually toward the right end by including NWs in contact, beginning with the leftmost NWs touching the left end of the simulation domain. If two paths share a conducting NW, those paths are agglomerated as a cluster to proceed in a single path. If a conducting path touches the right end of the domain, the corresponding NW agglomeration is found as a conducting cluster. The NWs that are not contained in the conducting cluster are discarded to construct an adjacency matrix. The adjacency matrix is constructed in a square matrix with each column and row associated with an individual contact point and the corresponding entry equal to either of contact and NW resistance.

Using the adjacency matrix, the overall resistance of the CNT network is calculated based on Kirchhoff’s current law (KCL) derived for the network with a 1V voltage source applied at both ends of the domain by considering an equivalent circuit network with two types of the resistance arising from inherent NW resistance and contact resistance between two NWs. KCL is applied at all contact points in the cluster to formulate a system of linear equations with respect to the voltage drop at every contact point in the conducting path. The solution of the system of linear equations, which can be obtained using linear algebra software packages. The total current flowing across the square domain is calculated from the solution. From the assumption of the 1V voltage source, the obtained total current corresponds to the overall conductance σ of the CNT network. The parameters of the NW considered in the simulation are set according to experimentally measured properties of currently available NWs. A NW is geometrically modeled as a cylinder with a diameter of 15 nm, the length of 5 μm, and the resistance of $2\times 10^{-2}\;\Omega$ [25]. The contact resistance for two NWs in contact is set to 500 Ω, considering current state-of-the-arts in fabrication technology [26]. The contact resistance is constant over the NW network since the tunneling resistance in the two-dimensional NW network does not undergo a large variation in the distance between NWs. The above procedure is repeated to obtain 500 independent random instances, and an ensemble average of the resistance is calculated based on the set of tested samples between 30th percentile and 70th percentile. The simulation is conducted over different configurations of silica content, silica size, CNT content, and CNT dimension. For massive simulation, the simulation is implemented in parallel processing package such MATLAB computing toolbox.

## 3. Results

**Figure 2:**
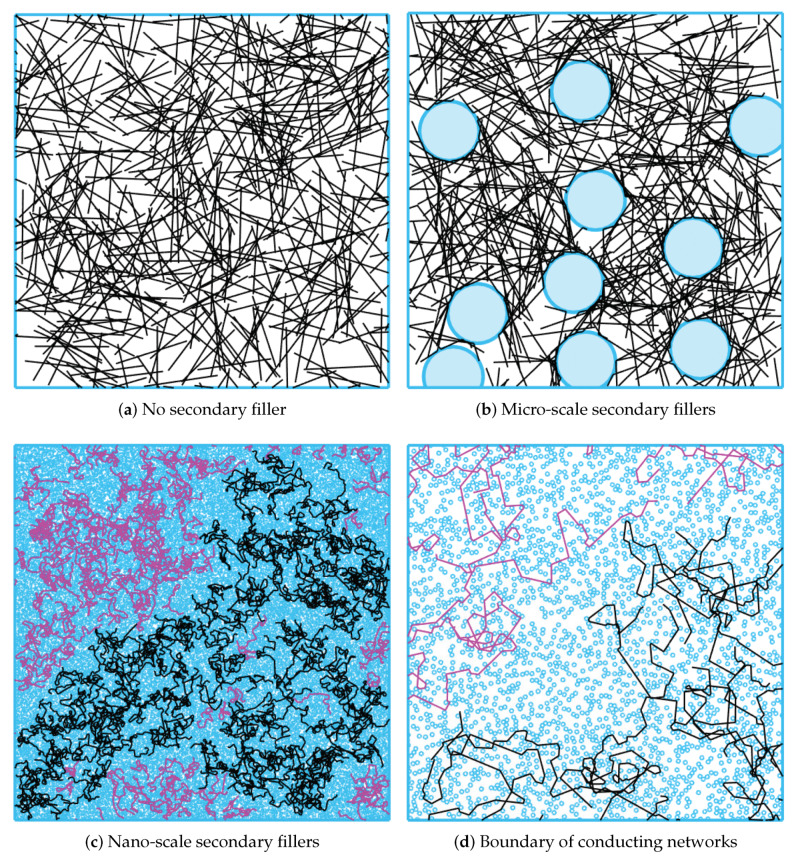
Random percolating networks for various cases.

The size effect of secondary particulate fillers on the electrical resistivity of the nanocomposites can be observed with random instances of the CNT/silica composites generated from the simulation presented in Figure 2. Figure 2a–c are percolating CNT networks of the same coverages of CNT NWs and particulate fillers for three cases of no secondary filler, micro-scale filler, and nano-scale fillers, respectively. A CNT network forming conducting paths is represented in black, while the remaining NWs isolated from the percolation network are represented in purple. Figure 2a shows a random network of CNTs with the diameter of 15 nm and the length of 5 μm in the simulation domain of 25 μm × 25 μm. In the simulation setup, the CNT content of 4% is dispersed in an epoxy resin. Since the percolation occurs in this configuration of the CNT network, all NWs are included in Conducting networks. By contrast, Figure 2b,c illustrate representative random instances of CNT/silica composites generated with the same loading amount of CNTs along with the micro-size (the diameter of 4 μm) and the nano-size (the diameter of 50 nm) particulate fillers, respectively, at the silica content of 20 wt%. In comparison with the case without secondary fillers, the micro-scale silica CNT composite network contains several dense CNT clusters formed by the excluded volume arising from the micro-size silica fillers, which is consistent with the results of existing studies about the excluded volume theory. Topological changes of the network, such as dense CNT clusters, lead to the enhancement in the chance that NWs in the domain CNTs are interconnected to form a large number of contacts. The total number of contact points in den CNT clusters forming major conducting paths in the percolating network increases from 502 to 2599 after incorporating micro-scale silica fillers. The increase in the number of contact points in the network evidences the improvement of the network electrical conductivity over nanocomposite systems without secondary fillers.

On the other hand, nano-scale secondary fillers prevent NWs from stretching toward the right side of the domain, and densely dispersed nano-sized particulate fillers cause the resulting system an abrupt increase with severe NW kinks. As shown in Figure 2c, the same loading amount of NWs covers only a fraction of the conducting network. The resulting topology change disrupts the formation of a conducting CNT network. The total number of interconnecting junctions in active percolating clusters decreases from 502 to 269. These results are consistent with the resistivity increase of the CNT/nano-size silica composite measured empirically. To see this in more detail, Figure 2d zooms in a boundary of conducting and nonconducting network regions in Figure 2c. In s micro-scale filler case, all regions unoccupied by two micro-scale particulate fillers between them, referred to as necks in percolation theory, have at least one NW passing. Regarding the percolation theory, a neck acts as a conducting path in the percolating network, and thus all necks participate in the conducting network. By contrast, only a few necks between nano-scale fillers have NWs passing through them, implying that a small number of necks act as a conducting path in the CNT network. Furthermore, the NW kinks incurred by the blockage of nano-scale fillers reduces the effective length of conducting NWs. Therefore, there is little chance that all NWs forms a conducting CNT network, and only a fraction of the two-dimensional domain has a conducting property, resulting in a large network sheet resistance of the network.

**Figure 3:**
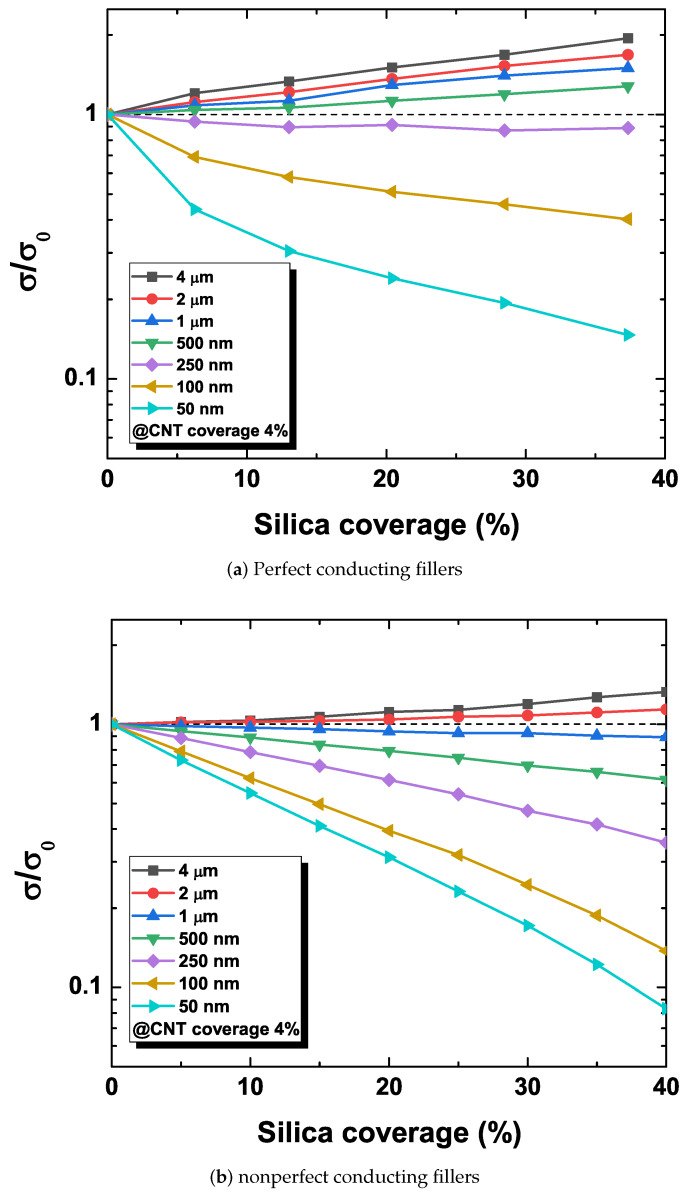
Normalized conductance changes for perfect and nonperfect conducting fillers with respect to silica coverage.

In order to assess a quantitative impact of the filler size, the electrical conductivity is monitored with varying secondary particulate filler contents with the CNT content fixed to 4%, which corresponds to the above electrical percolation threshold of the nanocomposite system. Figure 3 shows the change in the network sheet resistance for two cases of perfect conducting NWs and nonzero NW resistance. The change in the sheet resistance is represented by the ratio of the sheet conductivity without ($\sigma_{0}$) and with (σ) the secondary particulate fillers, i.e., $\sigma /\sigma_{0}$. Figure 3a shows the change of the conductivity of percolating networks consisting of perfect conducting NWs with zero resistance. In case of micro-scale particulate fillers, the increase in the micro-size filler loading content increases the electrical conductivity of the CNT network. According to the excluded volume theory, the improvement of the electrical conductivity relies on the excluded volume of secondary fillers driving concentrated NWs to create a dense CNT network. On the other hand, the incorporation of nano-scale silica fillers decreases the electrical conductivity as the silica filler content increases. This disruption of the CNT network degradation is attributed to the distortion of CNTs caused by dispersed nano-sized silica particles, and these conflicting behaviors caused by the excluded volume have been first explained with Voronoi geometry in [23]. Furthermore, the results reveal the existence of the secondary filler configuration that results in the invariance of the network conductivity with the change of the secondary filler coverage, corresponding to nanoparticle fillers with the diameter of 300 nm. Since such a point exists in any hybrid nanocomposite system consisting of multiple types of filler, this can be viewed as a criterion for comparison of the electrical conductivity performance.

Figure 3b shows the change of the conductivity of the CNT network with nonzero resistance nonperfect conducting NWs. This configuration shows similar trends of the change in the electrical conductivity, i.e., the conductivity ratio $\sigma /\sigma_{0}$ increases with increasing particulate filler sizes, whereas it reduces with the decreasing filler size with nano-scale particulate fillers, which are comparable to the corresponding conductivity change in the experimental data [23,27,28,29]. It is observed, however, that the slope of the conductivity change diminishes. The slope of the new system reflects a linear trend in conductance change with respect to the silica content in a log scale, as shown in experimental data [23]. Thus, the overall network conductivity is less sensitive to the contribution of the contact resistance. Furthermore, the size of particulate fillers leading to the conductivity invariance rises from 300 nm to 1.5 μm, which amounts to five times larger than the previous case. Thus, the network topology associated with the new case contains the same loading amount of NWs and secondary fillers but of a large scale. This is naturally expected since the consideration of additional resistance causes the increase of the overall resistance, leading to the decreased current. In order to compensate for the decreased current, additional conducting paths are necessary and can be achieved by allowing additional dense CNT clusters in the conducting network, which can be achieved with the increase in the secondary filler size. In fact, the value of overall network conductivity without secondary fillers $\sigma_{0}$ is increased.

**Figure 4:**
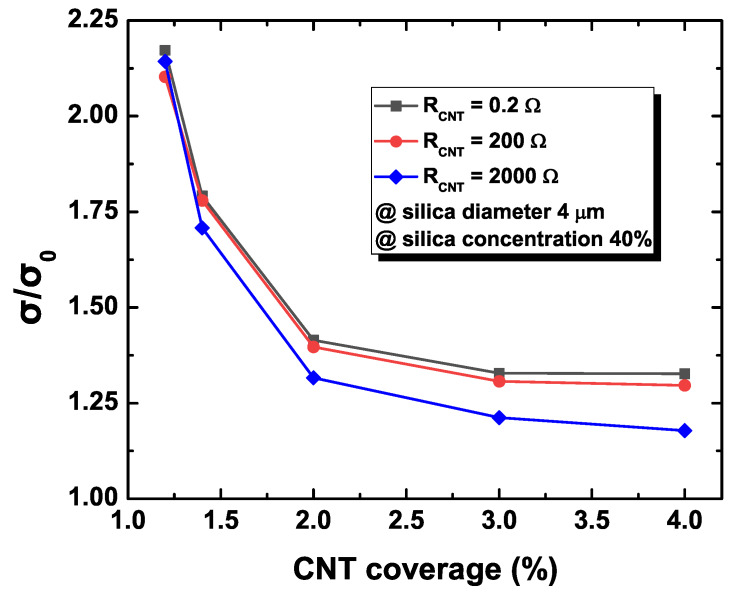
Normalized conductance changes with respect to NW coverage.

Figure 4 plots the changes in the sheet conductance of the percolating NW networks with respect to the value of the NW resistance denoted by $R_{CNT}$ in the secondary filler configuration of the diameter of 4 μm and content coverage of 40%. Random instances of the NW network considered in the simulation are basically identical except for the value of the NW resistance. In order to observe the degree of the difference, the normalized conductance change, defined as $\sigma /\sigma_{0}$, is measured to compare the average resistance of the composite system with and without silica fillers for increasing CNT contents under the fixed secondary silica content. The particulate filler effect on the conductivity of the composite system is maximized (or the most deviated from the original value) in low CNT loading regimes, while particulate fillers do not exhibit a significant impact in high CNT loading contents consistently for all cases of the NW resistance. The comparison among NW resistances indicates that the conductivity change is less sensitive for a large value of the NW resistance. The NW resistance and contact resistance contribute to the overall network resistance additively. In addition, the identical topology of the CNT networks leads to identical contact resistances for different configurations of NW resistance. This implies that the resulting normalized conductance change, defined as the ratio of the contact resistance contribution to the sum of the contact and NW resistance contributions, has an inversely proportional relationship with the value of NW resistance. This observation also reveals that the case with large contact resistance has the closest limit of the normalized conductance change toward one since the contribution of the NW resistance is minimized.

## 4. Discussion

This section provides the discussion about qualitative interpretations and underlying intuitions of quantitative results obtained from the MC simulation. A unified formalism is addressed for the analysis of the normalized conductivity change observed concerning the size of secondary particulate fillers. It can be established via the combination of discrete percolation theory and Swiss cheese model. Hybrid NW systems with particular secondary fillers can be viewed as a novel extension of Swiss cheese model. Swiss cheese model is indeed a porous medium percolation model where insulating particles occupy pores, while continuum such as fluid is allowed to flow for percolation. However, this nanocomposite system is nontrivial in that NWs which act as conducting paths which electrical current can flow through are overlaid in Swiss cheese model originally intended for the continuum percolation. In ordinary Swiss cheese model, the conductivity of a conducting path is characterized by the width of a neck, which is defined as a narrow path between surfaces of two neighboring spherical fillers. The conducting property of this system differs from ordinary Swiss cheese model, where the total conductance depends on the narrowest neck width since only a neck having conducting NWs can act as a valid conducting path. The density of NW clusters passing across the neck determines the electrical conductance of the associated neck. Thus, only a neck with nonzero conductance is a valid conducting path of the percolating network. For example, if a small number of NWs exist in the nanocomposite system, most necks do not contribute to the percolating network, resulting in low network conductance.

This framework is extended to the analysis of the nanocomposite system with micro- and nano-scale fillers. The nanocomposite system’s total conduct depends on two network parameters of the numbers of parallel conducting paths and the conductance of individual paths. A conducting path is formed by interconnecting active necks that contain conducting NWs so that it traverses the simulation domain, and the number of such conducting paths connected in parallel determines the amount of the total electrical current flowing across the domain. In addition, a conducting NW can be decomposed into two components in a horizontal direction toward the right end of the domain and its perpendicular vertical direction. The effective length of a conducting NW is defined as the length of the horizontal component of the conducting NW. A large effective length of conducting NW leads to a small number of contacts contained in a conducting path traversing the domain. The contact resistance of the resulting conducting path becomes small. On the other hand, the overall NW resistance does not differ among conducting paths since its value is proportional to the length of the conducting path, which is mostly similar to the length of the domain. Thus, the contact resistance has a dominant contribution to the overall conductance of a conducting path. The addition of micro-scale particulate fillers raises the density of conducting NWs along necks, improving the conductance of conducting paths, and increases the effective length of conducting NWs, leading to the reduction of the contact resistance of conducting paths. Therefore, the overall impact on the system is the increase in the conductance of the percolating network. The addition of nano-scale silica fillers, however, causes the decrease of the conductance of the percolating network, since nano-scale fillers block conducting NWs toward the end of the domain and make them twisted. The kinks of the conducting path increase the traversing distance across the domain, resulting in an increase in the NW resistance, and reduce the effective length of conducting NWs so that the contact resistance also grows. Note also that, since the addition of secondary fillers causes the increase and decrease of the network conductance, it is expected to exist a configuration of filler size that makes the conductance almost unchanged and can be estimated from computational results. Moreover, the aspects of the change in the network conductance for increasing silica content are also explained in a consistent way. According to the experiments and simulation results, the network conductance increases as the content of the micro-scale fillers increases. Additional content of the micro-scale filler increases the density of conducting NWs across necks, thereby increasing the number of current paths. For the increase of the nano-scale silica content, the network conductance, in contrast, diminishes as a large density of nano-scale fillers further increases the kinks of the NWs so that the effective length of conducting NWs shrinks. For the CNT/silica composites with silica fillers of a certain value of the diameter, the nanocomposite system’s total conductance remains almost fixed with increasing silica content. The effects of the introduction of additional current paths and the average conductance decrement are comparable, canceling out these opposite effects.

The consideration of nonzero NW resistance introduces several new behaviors of the electrical conductivity of the nanocomposite system. Nonzero NW resistance contributes to the total network resistance additively along with the contact resistance. Since the normalized conductance change is the ratio between the contact resistances, the sum of the contact resistance, and the NW resistance, its value is always less than one. The overall NW resistance indeed does not change very much since it depends on the length of the conducting path, which is given by the domain length and is mostly similar among conducting paths. On the other hand, the contact resistance of the nanocomposite system is created by the percolation phenomena. Furthermore, it normally has an exponential relationship with the secondary filler content. Meanwhile, the topology of two cases with and without nonzero NW resistance are identical, and the resulting contact resistance is also identical. Furthermore, the value of the NW resistance is relatively small as compared to the contact resistance, and the resulting normalized conductivity change is strictly less than one. For the increase of the silica content, the contribution of the NW resistance to the overall network conductance remains almost similar since there is little change in the effective length of the domain. By contrast, the contribution of the contact resistance to the network conductance grows since the addition of silica content changes the topology of the conducting network. Therefore, the resulting normalized conductance change becomes relatively small since the numerator and denominator both depend dominantly on the contact resistance.

**Figure 5:**
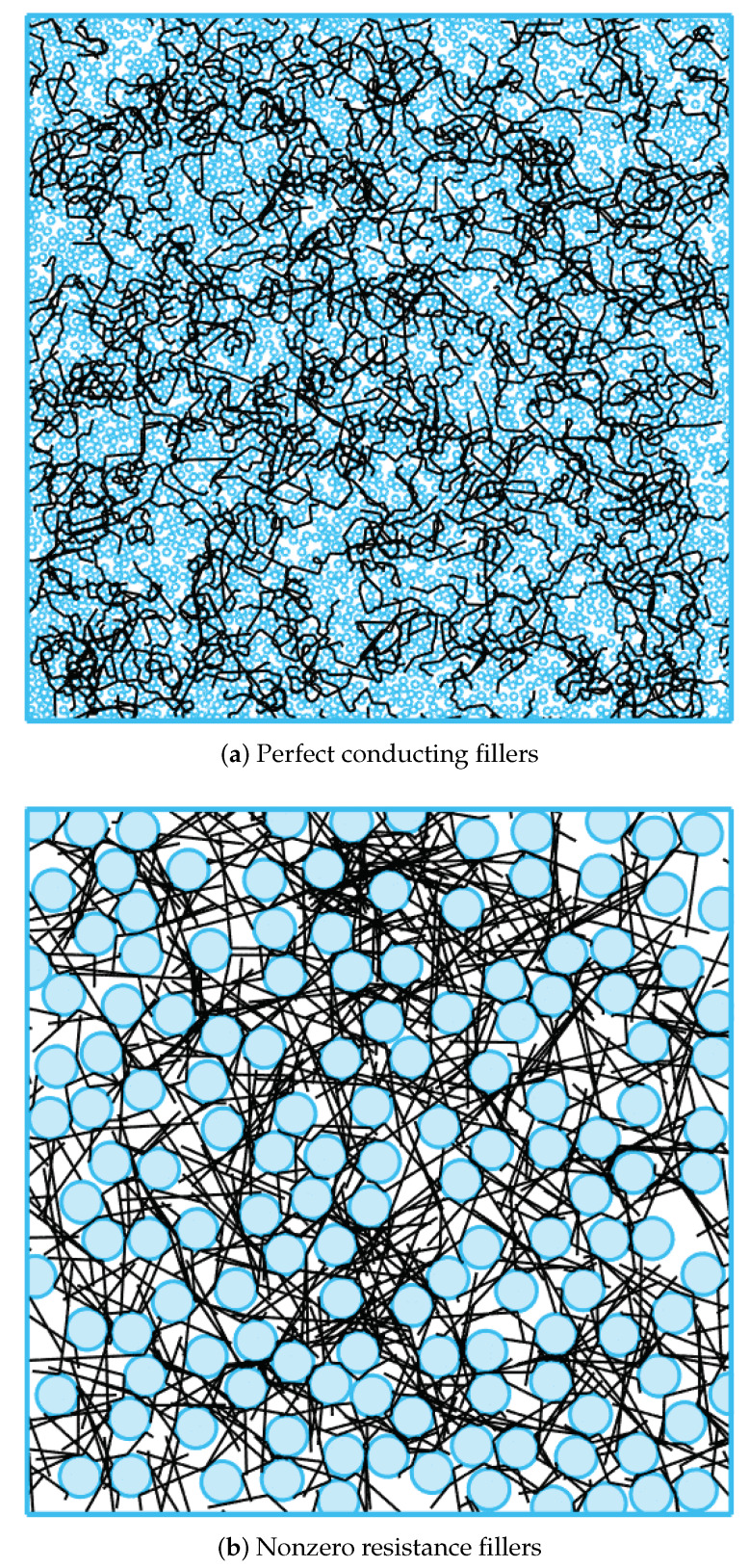
Random percolating configurations of conductance invariance with respect to secondary filler sizes.

Note here that the secondary filler configurations leading to invariant conductance are different. The case with nonperfect conducting NW requires a larger size of the particulate filler, as shown in Figure 5. In such a case, the overall conductivity decreases. The density of the conducting path needs to improve is to compensate the conductance decrease to obtain the same electrical performance as the case with perfect conducting NWs. Therefore, secondary fillers with large sizes are necessary, as in Figure 5b.

Finally, the discussion about the conductivity change caused by the CNT content change is ensured. Note that all configurations have the same geometrical topology with the percolating network. The nanocomposite system with larger WN resistance has a larger value of the normalized conductivity change, as in Figure 4. As the CNT content increases in the percolating network, the number of conducting paths increases, and the number of contact resistors that actually contribute to the percolating network decreases since additional CNT creates conducting paths with the reduced number of contact points. Therefore, the conductivity of the percolating network strictly depends on the contact resistance, which becomes gradually diminishing, and its limit approaches unity for increasing CNT content in the nanocomposite system.

In summary, we have shown that nonzero values of the electrical conductivity of NWs affect the conductivity variation of CNT composite networks containing secondary fillers along with the filler size. The overall network resistance increases via the addition of nonzero NW resistance, which has been ignored in the most of previous studies on the electrical property analysis of hybrid nanocomposite systems. In addition, the resulting slope of the change becomes less steep, in a linear change. Furthermore, the secondary filler configuration leading to an invariant network conductivity requires additional current so that it causes the increase with the filler size for nonzero NW resistance. The computational technique developed in this work can handle the electrical properties of comprehensive nanocomposite systems consisting of 1-D conducting wires and insulating particulate fillers such as silica-CNT, ZnO-CNT, and ${\mathrm{TiO}}_{2}$-CNT. These achievements shed light on practical design guidelines for the use of the secondary filler in the fabrication of highly-conducting and reinforced CNT composites.

## 5. Conclusions

This work has developed predictive computational models of the electrical conductivity of the two-dimensional random network with nonzero resistance conducting fillers and suggested strategies designed to enhance the electrical property. The electrical conductance is of a proportional relationship with the size of the secondary filler while showing a consistent decrease with the increasing NW coverage. The consideration of nonzero resistance NWs reduces the impact of the size of the secondary filler on the electrical conductivity of the percolating network, thereby causing linearized changes in the electrical conductivity of the network compared to the case of the perfect conductor. This relationship provides the resistance robustness of the overall network to allow a simplified guideline for the control of electrical properties for hybrid nanocomposite systems. For future research, this work can be extended to study the electrical property of three-dimensional bulk nanocomposites for computational characterization with improved accuracy.
